# Potent combination benefit of the AKT inhibitor capivasertib and the BCL-2 inhibitor venetoclax in diffuse large B cell lymphoma

**DOI:** 10.1038/s41375-024-02401-9

**Published:** 2024-09-16

**Authors:** Brandon S. Willis, Kevin Mongeon, Hannah Dry, India L. Neveras, Nadezda Bryan, Meghana Pandya, Justine Roderick-Richardson, Wendan Xu, Li Yang, Alan Rosen, Corinne Reimer, Liliana Tuskova, Pavel Klener, Jerome T. Mettetal, Georg Lenz, Simon T. Barry

**Affiliations:** 1 Bioscience, Early Oncology, AstraZeneca, Boston, USA; 2 Department of Medicine A, Haematology, Oncology, and Pneumology, University Hospital Münster, Münster, Germany; 3 Institute of Pathological Physiology, First Faculty of Medicine, Charles University Prague, Prague, Czech Republic; 4 Bioscience, Early Oncology, AstraZeneca, Cambridge, UK

**Keywords:** Preclinical research, Haematological cancer

## Abstract

The therapeutic potential of targeting PI3K/AKT/PTEN signalling in B-cell malignancies remains attractive. Whilst PI3K-α/δ inhibitors demonstrate clinical benefit in certain B-cell lymphomas, PI3K signalling inhibitors have been inadequate in relapsed/refractory diffuse large B-cell lymphoma (DLBCL) in part, due to treatment related toxicities. Clinically, AKT inhibitors exhibit a differentiated tolerability profile offering an alternative approach for treating patients with B-cell malignancies. To explore how AKT inhibition complements other potential therapeutics in the treatment of DLBCL patients, an in vitro combination screen was conducted across a panel of DLCBL cell lines. The AKT inhibitor, capivasertib, in combination with the BCL-2 inhibitor, venetoclax, produced notable therapeutic benefit in preclinical models of DLBCL. Capivasertib and venetoclax rapidly induced caspase and PARP cleavage in GCB-DLBCL *PTEN* wildtype cell lines and those harbouring *PTEN* mutations or reduced PTEN protein, driving prolonged tumour growth inhibition in DLBCL cell line and patient derived xenograft lymphoma models. The addition of the rituximab further deepened the durability of capivasertib and venetoclax responses in a RCHOP refractory DLBCL in vivo models. These findings provide preclinical evidence for the rational treatment combination of AKT and BCL-2 inhibitors using capivasertib and venetoclax respectively alongside anti-CD20 antibody supplementation for treatment of patients with DLBCL.

## Introduction

Diffuse large B-cell lymphoma (DLBCL) is the most common lymphoma subtype with approximately 30–40% of cases worldwide [1]. DLBCL is a heterogenous B cell malignancy defined by both genetic and epigenetic alterations that regulate cell growth, survival and differentiation [2]. Generally, DLBCL is transcriptionally subclassified into two major subset types, activated B-cell like (ABC) and germinal centre B-cell like (GCB) derived from distinct originating B cell populations. Genomic analyses have identified distinct subtypes of DLBCL with genetic drivers and signalling pathways that are therapeutically targetable [3]. First line therapy for DLBCL largely consists of rituximab, cyclophosphamide, doxorubicin, vincristine and prednisone (RCHOP), or more recently, a modified regimen substituting vincristine for polatuzumab vedotin (pola-R-CHP) [4]. Despite these recent improvements in first line therapies, relapsed/refractory DLBCL patients still require novel treatment regimens.

Activation of the canonical phosphoinositide-(3)-kinase (PI3K), AKT and mammalian target of rapamycin (mTOR) signalling pathway is frequently dysregulated across B-cell malignancies [5]. There are frequent mutations found in *PIK3CA* and *PTEN*, including down-regulation of PTEN expression in GCB DLBCL [6]. PI3K-AKT pathway inhibitors show differential activity in ABC- versus GCB-DLBCL subclassifications. ABC-DLBCL, which harbour PIK3CA mutations, is sensitive to PI3Kα and PI3Kδ inhibitors [7–9]. Copanlisib, a pan-PI3K inhibitor with PI3Kα and δ activity has clinical activity in follicular lymphoma and has been evaluated in ABC-DLBCL [10, 11]. Safety concerns associated with long-term treatment limits the utility of PI3K inhibitors such as copanlisib and idelalisib. Modulation of the PI3K signalling axis at distinct pathway nodes such as AKT is feasible using the small molecule pan-AKT inhibitor, capivasertib, which has an acceptable safety profile and shown positive benefit in a Phase III clinical trial and FDA approval in combination with fulvestrant for the treatment of hormone receptor positive breast cancer [12].

Relapsed/refractory GCB-DLBCL has a high incidence of PTEN protein loss [6], and GCB DLBCL cell lines and tumour models are sensitivity to AKT inhibition [7, 13], underscoring the importance of the PI3K-AKT signalling axis in GCB-DLBCL. This is further supported by the observation that combined PI3Kβ/δ and mTORC1/2 inhibition has broad efficacy across preclinical DLBCL models [14]. PI3K-AKT signalling pathways in DLBCL are therefore essential in different subtypes whilst the key pathway node for therapeutic modulation varies amongst subtypes.

R-CHOP is the conventional treatment for patients with DLBCL, along with the recent approval of Pola-R-CHP [4]. Additionally, clinical benefit in patients with various haematological malignancies has been observed using the B-cell lymphoma-2 (BCL-2) inhibitor venetoclax which induces apoptosis [15]. Venetoclax is approved for newly diagnosed and relapse/refractory adult patients with chronic lymphocytic leukaemia (CLL)/small lymphocytic lymphoma with 17p deletion, and for newly diagnosed acute myeloid leukaemia patients in combination with various hypomethylating agents [16, 17]. In Phase II clinical trial, addition of venetoclax to RCHOP enhanced clinical benefit in high risk DLBCL patients [19]. Preclinically, venetoclax combined with an epigenetic EZH2 inhibitor extended survival benefit of DLBCL PDX mouse models [18, 19]. Finally, venetoclax resistance in DLBCL cell lines associates with AKT signalling activation, suggesting that AKT antagonism could improve venetoclax response [20–23].

Given PI3K-AKT dependency in DLBCL, combination of capivasertib and venetoclax along with an anti-CD20 antibody, rituximab could present a unique therapeutic opportunity for patients with relapse/refractory DLBCL [24–27]. Herein, we provide preclinical evidence of capivasertib and venetoclax combinations along with the addition of rituximab as an attractive, novel treatment regimen for treatment of GCB DLBCL. Specifically, this combination rapidly activates caspases leading to robust tumour cell death driving anti-tumour efficacy in preclinical models of relapse/refractory DLBCL.

## Methods

### Combination screen

The combination of capivasertib and venetoclax was evaluated in an in vitro DLBCL cell panel proliferation screen as previously published [28]. Cells were seeded between 1000–8000 cells per well in 384-well plate. 24 h after plating cells were treated for 120 h with a 6-point dose response (0.03–3 µM) in a combination matrix (6 × 6) using an Echo 555 acoustic dispenser (Labcyte). Live cells were quantified using an imaging-based assay utilising Sytox Green (ThermoFisher, MA) and saponin (Sigma–Aldrich, MO) to permeabilise the cells. Cell proliferation versus cell death was determine by comparing Day 0 (pre-treatment) and 72 or 120 h (post-treatment). Percentage growth was calculated from the live cell number. Two-dimensional dose response matrix and curve fitting were processed in the combination extension of Genedata Screener 13™ (Genedata AG, Basel,Switzerland) as previously described [28]. Combination activity was calculated using the Loewe dose-additivity model using a synergy score cut-off >5.

### Cell line culture and reagents

DNA fingerprinting short tandem repeat authentication of cell lines was performed. Culture media are listed in supplementary Table S1. In caspase-glo 3/7 (Promega, WI) experiments, cells were pre-treated overnight with Q-VD-OPH (50 µM) (Cayman Chemical, MI) or vehicle prior to seeding. Capivasertib (AZD5363) was synthesized at AstraZeneca. Venetoclax was purchased from Medkoo Biosciences (Durham, NC). Generation of stable genetic knockout cell lines using Ribonucleoprotein (RNP) nucleofection was conducted using the NEON electroporation system (Invitrogen, CA). Details on the sgRNAs are captured in supplementary Table S3.

### Biomarker analysis

Western blots were performed as previously described [29]. Cells were lysed in Phosphosafe lysis reagent (EMD Millipore, MA) supplemented with 1X HALT Protease and Phosphatase Inhibitor (PPI) Cocktail (Thermo Fisher, MA). A 1% (w/v) SDS, 10% (v/v) glycerol, 0.1 M Tris-HCl (pH 6.8) lysis buffer supplemented with 1:1000 benzonase (Sigma Aldrich, MO) and HALT PPI cocktail was used for protein extraction to probe for BAK and BAX. Horseradish peroxidase–linked secondary antibodies (Cell Signalling Technology, MA) and DETECTED Pierce ECL SuperSignal™ West Pico PLUS Chemiluminescent Substrate or Femto Maximum Sensitivity Substrate (Thermo Fisher, MA) were used to detect immune complexes. Cytochrome c release assay kit was performed according to manufacturer instructions (Abcam, UK). Primary antibodies details are shown in Supplementary Table S2.

### In vivo studies ethics approvals

All cell line models were conducted in accordance with the AstraZeneca IACUC (Institutional Animal Care and Use Committee) and reported following the ARRIVE (Animal Research: Reporting In Vivo experiments) guidelines [30]. PDX studies involving the use of laboratory animals conducted in the Center for Experimental Biomodels of the First Faculty of Medicine, Charles University in Prague, Czech Republic, were reviewed and approved by the Institutional Animal Welfare Committee and by the Ministry of Agriculture of the Czech Republic, as well as by the Research and Higher Education section of the Ministry of Education, Youth and Sports of the Czech Republic under the number 592/15 (MSMT-11255/2015-4).

### In vivo studies

Cell line derived models were powered according to the AstraZeneca powering tool depending on degree of anti-tumour response anticipated. For PDX models a minimal of 8 animals per group was used. Studies were performed independent of the lead investigators, though compound identity was not blinded. No animals were excluded. Animals were randomized to a consistent mean tumour size per group. All calculations were performed using AstraZeneca. Tumour volume was calculated according to the following formula: π/6 × length × width × height). Statistical analyses. Data shown in graphs are mean ± SEM. Statistical analyses (t-test, multiple comparisons) were performed using GraphPad Prism (version8.4.3). Mouse xenograft models of DLCBL cell lines. Ten million SuDHL4 and WSU-DLCL2 or five million SUDHL5 cells in PBS mixed with 50:50 with Matrigel™ (Beckton Dickinson, Franklin Lakes, NJ, USA) were implanted subcutaneously into the right flank of CB-17 *scid* female mice. For efficacy studies, growth inhibition from the start of treatment was assessed by comparison of the differences in tumour volume between control and treated groups. Statistical significance was evaluated using a one-tailed, 2-sample *t* test. Capivasertib was formulated as an oral solution in 10% DMSO/25% Kleptose and dosed 130 mg/kg BID. Venetoclax was formulated as an oral suspension in 60% Phosal 50 PG, 30% polyethylene glycol 400, 10% ethanol. A 4 h gap was provided between each agent with capivasertib dosed first. Long term administration of venetoclax in combination with capivasertib was associated with body weight loss. Dosing holidays were provided and food supplementation when body weight loss was greater than 10–12% for an individual mouse. Body weight loss is because of glucose dysregulation is a common mouse toxicity following inhibition of PI3K-AKT signalling. RCHOP was administered once; Rituxan® (rituximab) 10 mg/kg intraperitoneal, Cytoxan® (cyclophosphamide) 25 mg/kg intraperitoneal, Doxil® (doxorubicin hydrochloride) 3 mg/kg intravenous, Oncovin® (vincristine sulfate) 0.25 mg/kg intravenous, Deltasone® (prednisolone) 0.5 mg/kg by oral gavage, each according to manufacturer’s instructions. Patient Derived Xenograft mouse model of DLBCL. For EBV-negative PDX models, six- to eight-week-old female NOD.Cg-Prkdc severe combined immunodeficiency Il2rgtm1Wjl/SzJ (NSG; Jackson Laboratory, ME, USA) mice were used.

## Results

### Combined AKT and BCL-2 inhibition is effective in wildtype and PTEN-deficient DLBCL cell lines

**Fig. 1:**
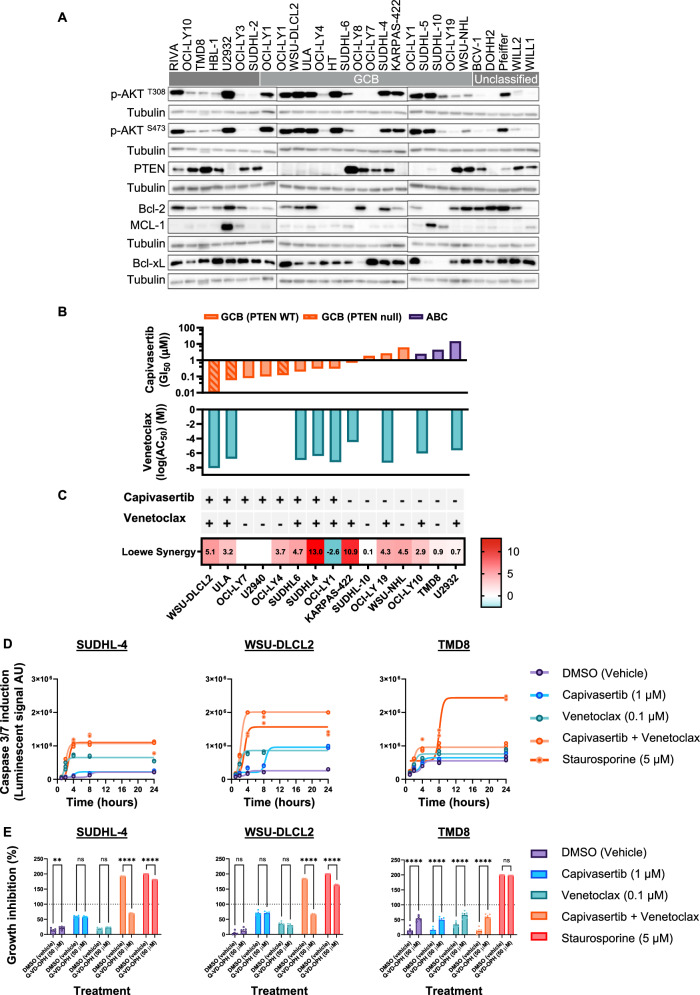
Capivasertib and venetoclax combination results in rapid caspase-mediated cell death in responsive DLBCL models. **A** Base line Western blot biomarker profiling of ABC-, GCB-, and unclassified-DLBCL cell models with the OCI-LY1 cell line serving as reference control. **B** Capivasertib monotherapy anti-proliferative activity (GI_50_) (μM) and venetoclax monotherapy apoptotic response (Log AC_50_) (M) across GCB- and ABC-DLBCL cell lines. Order of cell lines is identical to that shown at the bottom of (**C**). **C** Loewe synergy scores for the combination of capivasertib and venetoclax in GCB- and ABC-DLBCL cell lines. + and – indicates each cell line’s monotherapy sensitivity to either capivasertib or venetoclax. Order of cell lines identical to that shown in (**B**). **D** Time course of caspase-3/7 activity in a cell model unresponsive to the either monotherapy or combination (TMD8), and two responsive models: WSU-DLCL2 (PTEN-mutant; GCB) and SUDHL-4 (PTEN wild-type; GCB) (*n* = 3/group). **E** Relative cell proliferation of SUDHL4, WSU-DLCL2 and TMD8 cell lines pretreated overnight with the pan-caspase inhibitor Q-VD-OPH (50 µM) or vehicle and then dosed with compounds for 24 h (*n* = 5/group; 2-way ANOVA with Sidak’s multiple comparisons ****p < 0.0001). Staurosporine (5 µM) was a positive control for caspase induction and cell killing.

Capivasertib monotherapy treatment is effective in GCB DLBCL cell lines and xenograft models [7]. In an attempt to deliver a deeper anti-tumoral response, the activity of capivasertib and venetoclax was explored across a panel of 26 DLBCL cell lines. The panel of 7 ABC, 14 GCB, and 5 unclassified DLBCL cell lines had heterogenous levels of AKT pathway activation, PTEN protein and anti-apoptotic cell death proteins BCL-2, BCL-2-like 1 (BCL-XL) and MCL-1 expression (Fig. 1A). Due to limits in screening capacity, representative lines that capture the DLBCL landscape were selected for a 3-day combination proliferation screen. Monotherapy anti-proliferative responses (GI_50_) with capivasertib were enriched in PTEN deficient cell lines whereas venetoclax monotherapy resulted in cell death (AC_50_) in GCB-DLCBL and ABC-DLCBCL subtypes (Fig. 1B). Capivasertib and venetoclax exhibited strong combination activity (determined by Loewe score), predominantly in the GCB-DLCBL subcategory regardless of PTEN status (Fig. 1C), although certain pAKT and BCL-2 positive lines were insensitive to the combination.

To investigate changes following combination treatment, sensitive PTEN-wild-type (SUDHL-4) and PTEN-deficient (WSU-DLCL2) GCB-DLBCL, as well as an insensitive PTEN wild-type (TMD8, OCI-LY10, U2932), ABC-DLBCL cell lines were evaluated (Fig. 1D, Supp. Fig. 1A). Combination treatment induced rapid induction of caspase 3/7 within 4 h, which was maintained at 24 h in SUDHL-4 and WSU-DLCL2 cell lines whereas the insensitive PTEN wild type cells lacked caspase induction (Fig. 1D, Supplementary Fig. 1A). Interestingly in the PTEN-deficient U2932 cell line whilst combination treatment did not increase caspase 3/7 induction, over long-term treatment a combination benefit was observed, with reduced proliferation versus both monotherapy treatments suggesting acute induction of apoptosis is not necessary for combination benefit (Supp. Fig. 1B). Pharmacological rescue of sensitive GCB-DLBCL cell lines with Q-VD-OPH, a pan-caspase inhibitor, confirmed caspase-mediated cell death (Fig. 1E). In general, the combination induced extensive cell death within 24 h, suggesting that targeting AKT and BCL-2 has potential to enhance DLBCL tumour cell death.

### Capivasertib and venetoclax combine to induce caspase-dependent cell death through BAK and BAX

**Fig. 2:**
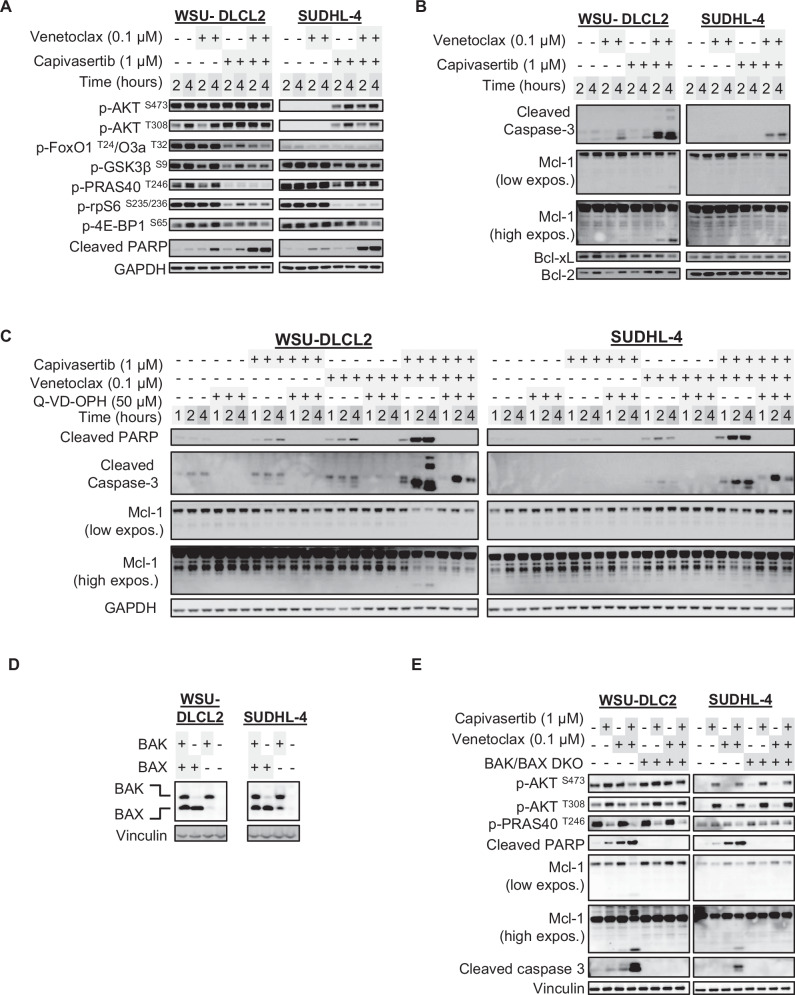
Combination-mediated induction of cell death markers ablated by BAK/BAX codeletion in responsive DLBCL cell models. **A** Western blot profiling of the AKT signalling pathway, known AKT substrates, and (**B**) apoptotic markers in WSU-DLCL2 and SUDHL-4 when treated with capivasertib and venetoclax monotherapies and in combination at 2and 4 h. **C** Western blot profiling of apoptotic biomarkers time course of WSU-DLCL2 and SUDHL4 cell lines pretreated with vehicle (DMSO) or Q-VD-OPH (50 µM) then dosed with indicated compounds at 1, 2, and 4 h. **D** CRISPR/Cas9-mediated of single and double knockout (DKO) of BAK and BAX in WSU-DLCL2 and SUDHL4 cell lines. **E** Comparison of the induction of cell death markers between BAK/BAX wild-type and DKO WSU-DLCL2 and SUDHL4 cell lines when treated with compounds for 4 h.

Next, biomarker changes associated with enhanced cell death in the SUDHL-4 and WSU-DLCL2 cells were evaluated. Consistent with the capivasertib mode of action, AKT phosphorylation is enhanced followed by a reduction in phosphorylation of AKT substrates (Fig. 2A) [31–41]. Despite elevated basal AKT activation in PTEN-deficient WSU-DLCL2 cells, capivasertib reduced phosphorylation of S6 kinase in both WSU-DLCL2 and SUDHL-4 cells (Fig. 2A) indicating suppression of PI3K-AKT axis. Modulation of other substrates GSK3β and PRAS40 differed between cell lines indicating downstream signalling heterogeneity. Alterations in phosphorylation of FOXO and 4EBP1 were consistent with inhibition of AKT signalling (Fig. 2A). Venetoclax monotherapy induced minimal PARP1 and caspase-3 cleavage in both WSU-DLCL2 and SUDHL-4 cell lines (Fig. 2A, B). Following combination treatment modulation of AKT signalling emulated capivasertib monotherapy (Fig. 2A). Capivasertib monotherapy had minimal effect on BCL-2 and BCL-XL protein levels or apoptotic biomarkers however, the combination increased PARP1 and caspase-3 cleavage in both GCB-DLBCL cell lines. At certain time points there were reductions in MCL-1 protein levels and MCL-1 cleavage in the SUDHL4 and WSU-DLCL2 cell line however these changes were variable and hence further work is required to determine whether these MCL changes make a contribution to efficacy (Fig. 2B, C) [42, 43]. In the SUDHL4 and WSU-DLCL2 cell lines, combination treatment led to an enhancement in cytochrome c release into the cytoplasm relative to either monotherapy treatments (Supplementary Fig. 2A). As expected, pre-treatment with Q-VD-OPH inhibited combination apoptosis induction (Fig. 2C).

**Fig. 3:**
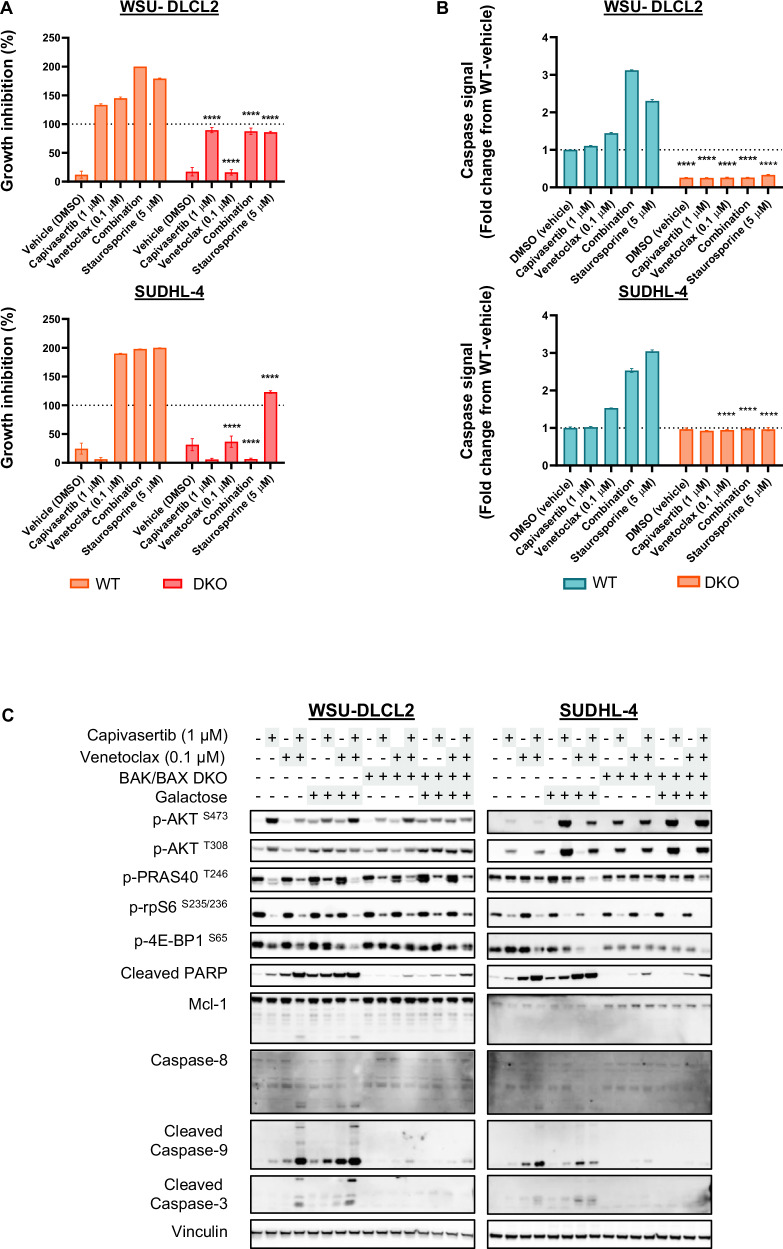
Combination-mediated induction of cell death markers ablated by BAK/BAX codeletion in responsive DLBCL cell models. **A** Growth inhibition of BAK/BAX WT and DKO WSU-DLCL2 and SUDHL4 cells treated as indicated in a 72-hr measured by relative Cell Titer Glo signal growth assay (*n* = 3/group; 2-way ANOVA with Sidak’s multiple comparisons of the means between WT and DKO in the same treatment group ****p < 0.001). **B** Relative fold change of caspase-8 activity in BAK/BAX WT and DKO WSU-DLCL2 and SUDHL4 cells treated as indicated for 4 h. Staurosporine (5 µM) was a general positive control for caspase induction and cell killing. *n* = 3/group; 2-way ANOVA with Sidak’s multiple comparisons of the means between WT and DKO in the same treatment group ****p < 0.001. **C** Western blot profile of BAK/BAX WT and DKO WSU-DLCL2 and SUDHL4 cells treated with compounds for 4 h in normal glucose-rich growth medium or with medium where glucose is substituted with galactose.

Canonical apoptosis induction occurs through mitochondrial outer membrane pore (MOMP) formation with caspase activation preceding PARP1 cleavage and MCL-1 degradation (reviewed in [44–47]). To confirm canonical apoptotic pathway mechanisms, CRISPR/Cas9 gene editing was used to generate BAK and BAX deficient WSU-DLCL2 and SUDHL-4 cell lines with individual and tandem knock out of each gene s (Fig. 2D) [48–50]. Combination treatment of BAK/BAX double knockout (DKO) cells did not induce apoptosis at 4 h, whilst capivasertib reduced AKT signalling (Fig. 2E, Supplementary Fig. 2B). Growth inhibition in BAK/BAX DKO GCB-DLBCL cell lines following combination treatment was equivalent to monotherapy capivasertib (Fig. 3A). Individual knockdown of either BAX or BAK did not block induction of apoptosis (Supplementary Fig. 2B) suggesting cell priming death is mediated through BAX or BAK. Additionally, caspase-8 induction was attenuated in the BAK/BAX DKO GCB-DLBCL cell lines after combination treatment (Fig. 3B, C). These results suggest that combining capivasertib and venetoclax induces cell death by direct effects on mitochondria.

Given AKT signalling regulates metabolism and in particular glucose homeostasis, we hypothesised that metabolic stress may enhance venetoclax activity. We evaluated whether galactose substitution in media could mimic capivasertib mediated inhibition of glucose uptake. Galactose supplementation failed to modulate AKT pathway activation or inhibition of AKT signalling by capivasertib in wildtype and BAK/BAX DKO cell lines. Interestingly, in galactose-substituted medium venetoclax monotherapy treatment resulted in greater induction of cell death markers than in glucose-rich medium, but induction of cell death was not as effective as capivasertib and venetoclax (Fig. 3C). Moreover, cleavage of caspases -3, and -9 was enhanced by combination relative to venetoclax treatment irrespective of medium conditions (Fig. 3C). Again, combination treatment induced cleavage of caspase-9, -3 and -8 (a component of the death-inducing signalling complex canonically associated with the extrinsic apoptosis pathway) and was abrogated upon deletion of BAK and BAX in the presence and absence of galactose (Fig. 3C). Collectively, these data suggest that whilst modulation of glucose uptake and metabolic regulation by capivasertib could enhance apoptosis induction by venetoclax, other AKT dependent mechanisms are contributing to the combination effect.

### Capivasertib and venetoclax are efficacious in wildtype and PTEN-null DLBCL tumour xenografts with different doses and schedules

**Fig. 4:**
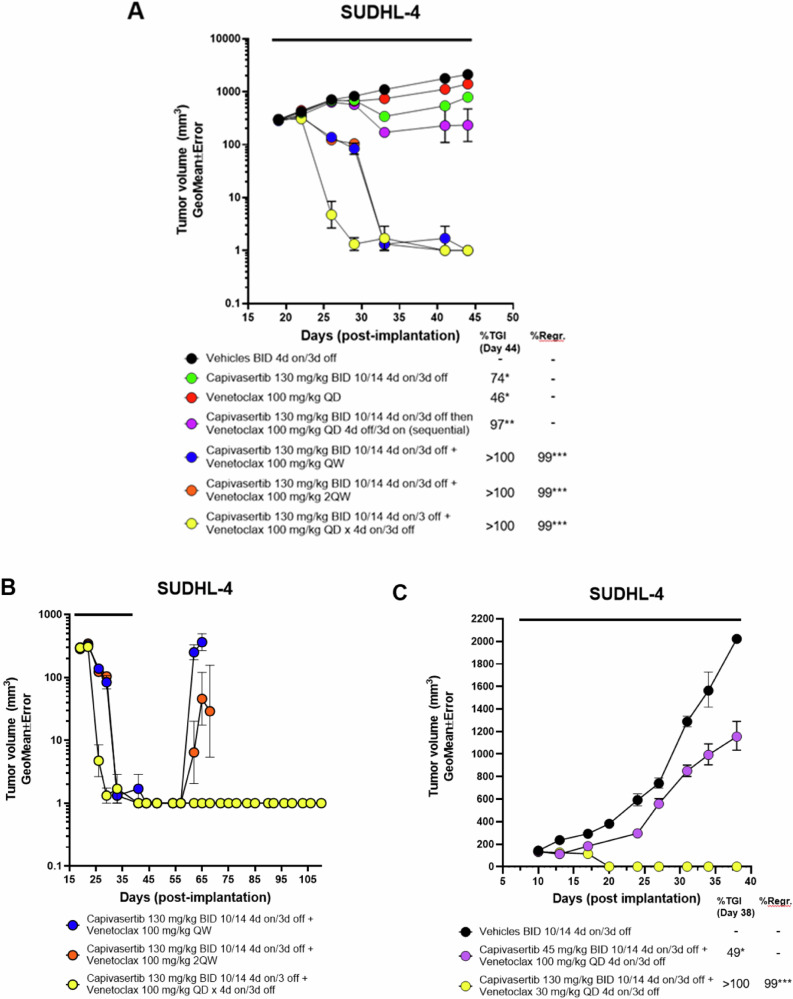
Combination of capivasertib with venetoclax induces sustained tumour regression in a PTEN-wildtype SUDHL4 xenograft model. **A** Tumour growth curves of CB.17 SCID mice bearing the GCB-DLBCL cell line SuDHL4 xenograft tumours treated with capivasertib (130 mg/kg BID 10/14 4-day on/3-day off) monotherapy, venetoclax (100 mg/kg QD) monotherapy and in combination in accordance with schedules captured in Supplementary Table 1. **B** Assessment of sustained tumour regression (>60 days post dosing). Both capivasertib and venetoclax were administered on a 4-day on/3-day off schedule relative to combinations using reduced frequency of venetoclax treatment. **C** Assessment of tumour activity of capivasertib dose reduction versus venetoclax dose reduction on anti-tumour response or tumour regression. Data is represented as geometric mean of tumour volumes and standard error of the mean. *p < 0.05, **p < 0.001, ***p < 0.0001.

Next the capivasertib and venetoclax combination was validated in vivo. Clinically, capivasertib is administered using an intermittent 4-days on and 3-days off schedule and venetoclax once daily. Consideration of needing to alter dose and schedule in mice with this combination, alternative schedules to evaluate efficacy in vivo was also explored. Mice bearing *PTEN*-wildtype SUDLH-4 tumour xenografts were treated with both capivasertib 130 mg/kg BID 4-days on/3-days off and venetoclax 100 mg/kg once daily 4-days on/3-days off. Both capivasertib or venetoclax monotherapy treatments gave modest inhibition of relative tumour growth (74% and 46% TGI respectively). Combination treatment gave complete tumour regression (Fig. 4A). Whilst concurrent dosing was highly efficacious, combination treatment on a 4-days on/3-days off schedule exhibited occasional but manageable body weight loss in mice. Therefore, considering that frequent dose alterations of venetoclax are provided clinically, exploration of different dose schedules the combination were evaluated to understand different combination regimens that maintained efficacy whilst minimising potential tolerability issues (Supp. Table S4 and Supplementary Fig. 3A, B). Doses of both drugs varied, however the 4-days on/3-days off capivasertib schedule remained constant whilst frequency of venetoclax varied. Sequential dosing of capivasertib for 4 consecutive days followed by venetoclax for 3 consecutive days reduced combination benefit showing concurrent drug administration drives optimal efficacy (Fig. 4B). Reducing either the dosing frequency or dose level of venetoclax whilst maintaining the clinically relevant capivasertib treatment regimen was assessed. Reduction venetoclax from 100 to 30 mg/kg once daily 4-days on/ 3-days off concurrently with capivasertib drove tumour regressions (Fig. 4C). However, duration of tumour response after treatment cessation was reduced versus the full clinically relevant schedule (data not shown). Frequency reductions of venetoclax administration to 100 mg/kg once daily to day 1 or days 1 and 3 drove tumour regressions with minimal body weight loss (Fig. 4A, B and Supplementary Fig. 3A). However, tumour regrowth was observed 10 days after dosing cessation. Finally, reducing capivasertib to 45 mg/kg 4-days on/3-days off schedule with 100 mg/kg of venetoclax led to a loss of efficacy (Fig. 4C, Supplementary Fig. 3C). Collectively, this suggests that inhibition of AKT is sufficient in priming the combination response, however the induction of cell death can still be achieved with various dose levels of both compounds and frequency of venetoclax treatment.

### Combination treatment reduces growth of human DLBCL PDX models

**Fig. 5:**
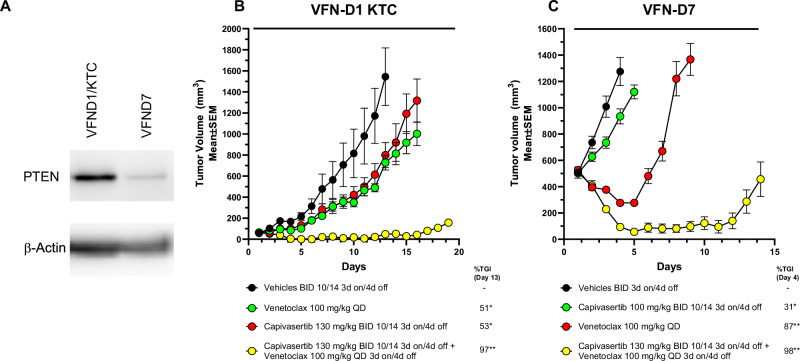
Combination of capivasertib with venetoclax promotes tumour regression in a PTEN-wildtype and PTEN-null DLBCL PDX mouse models. **A** Western blot analysis of PTEN expression in the VFN-D7 and VFN-D1_KTC human DLBCL PDX models. **B** Tumour growth curves of NSG mice bearing tumours with the double-hit GCB DLBCL PDX model, VFN-D7 or (**C**) the non-GCB DLCBL PTEN deficient PDX model, VFN-D1_KTC treated with capivasertib on a slightly modified schedule (130 mg/kg BID 10/14 3-day on/4-day off) monotherapy, venetoclax (100 mg/kg QD) and in combination with both agents on 3-day on/4-day off schedule. Comparison of combination treatment tumour regression compared to either monotherapy groups. Data is represented as arithmetic mean and standard error of the mean. *p < 0.05, **p < 0.001, ***p < 0.0001.

To further explore this combination in vivo, two DLBCL PDX mouse models that represent relapsed/refractory DLBCL were evaluated. A GCB PDX model, VFN-D7 DHL with low PTEN protein (Fig. 5A) expression harbours both a BCL2 and MYC translocation, hallmarks of double hit lymphoma and the VFN-D1 KTC PDX model has wildtype PTEN protein levels (Fig. 5A), a BCL2 amplification and MYC copy number gain. PDX models were derived from chemotherapy refractory DLBCL patients [51, 52]. These PDX models are established in NSG mice and therefore to accommodate differences in tolerance amongst mouse strains, the drug combination was administered on a modified schedule of 3-days on/4-days off (Supplementary Fig. 3D, E). In both models, the capivasertib and venetoclax combination suppressed tumour growth. (Fig. 5B, C). Therefore, the combination can also deliver improved anti-tumour benefit in vivo in BCL2-positive chemotherapy refractory DLCBL malignancies.

### AKT and BCL-2 inhibition combines with CD20 antagonism and overcomes RCHOP resistance

**Fig. 6:**
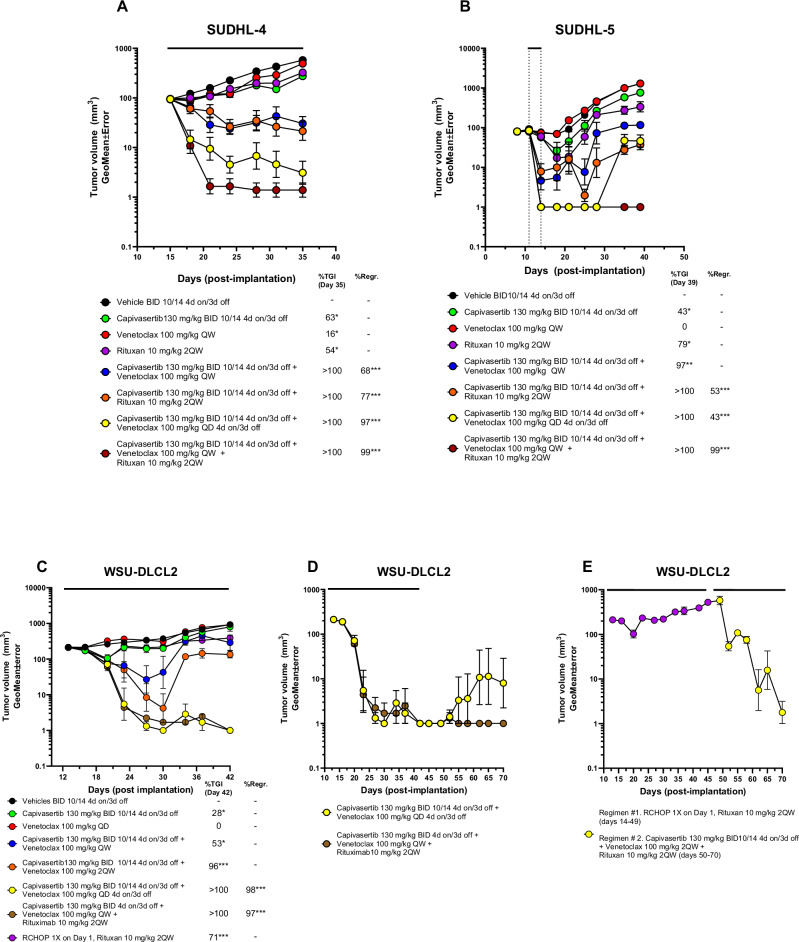
Combination of capivasertib, venetoclax and rituximab promotes tumour regression in GCB-DLBCL cell line xenograft models and in a RHCOP resistant DLBCL xenograft model. Assessment of tumour regressions following addition of once weekly rituximab to capivasertib and venetoclax relative to the capivasertib and venetoclax combination on 4-day on/3-day off schedule. **A**, **B** Tumour growth curves of CB.17 SCID mice bearing with the GCB-DLBCL cell line (**A**) SUDHL4 and (**B**) SUDHL5 xenograft models treated with capivasertib (130 mg/kg BID 10/14 4-day on/3-day off) monotherapy, venetoclax (100 mg/kg QD) monotherapy or rituximab (10 mg/kg 2QW) monotherapy and in combination in accordance with schedules shown in Supplementary Table 1. **C** Comparison of initial tumour response and tumour regression following the triple combination of capivasertib, venetoclax and rituximab in the WSU-DLCL2 xenograft model relative to the doublet combinations and RCHOP (10 mg/kg rituximab 2QW, 25 mg/kg cyclophosphamide once IP, 3 mg/kg doxorubicin hydrochloride once IV, 0.25 mg/kg vincristine sulphate once IV and 0.5 mg/kg prednisone QD5 PO) treated as indicated (see Supp. Table S4). **D** Assessment of the sustained tumour regressions in the capivasertib venetoclax versus capivasertib venetoclax rituximab groups. **E** Tumours from the RCHOP treated group shown in (**C**) that were resistant to treatment (purple dots) were retreated with the capivasertib, venetoclax and rituximab triplet (yellow dots) and tumour regression assessed. All data is represented as geometric mean of tumour volumes and standard error of the mean (*p < 0.05, **p < 0.001, ***p < 0.0001).

Next, we evaluated whether antagonising CD20 using rituximab could further the enhance combination response in PTEN-wildtype SUDHL4, PTEN low protein SUDHL5, and *PTEN*-null WSU-DLCL2 xenograft models. The triple regimen consisted of capivasertib on 4-day/3-day off schedule, venetoclax given twice weekly and rituximab given twice weekly. In the SUDHL4 xenograft model, the triple combination of capivasertib, venetoclax and rituximab achieved superior efficacy relative to monotherapy and combination arms (Fig. 6A, Supplementary Fig. 3F). Similarly, superior, continuous efficacy was observed in the SUDHL5 xenograft model after a single week following the triple drug regimen exemplifying the anti-tumoral response of this combination (Fig. 6B, Supplementary Fig. 3G). In WSU-DLCL2 xenograft model, the triple regimen resulted in long-term durable responses relative to the combination capivasertib and venetoclax (Fig. 6C, D, Supplementary Fig. 3H). Lastly, given that most DLBCL patients receive RCHOP therapy, we sought to determine whether the triple regimen has potential to be remain active in tumours progressing on RCHOP therapy. Mice bearing WSU-DLCL2 tumours were treated with RCHOP then during tumour progression, mice received the triple regimen which led to rapid tumour regression with kinetics similar to RCHOP naïve DLBCL tumour xenograft models (Fig. 6E, Supplementary Fig. 3J). Collectively, these data suggests that treatment with capivasertib and venetoclax can be further enhanced with anti-CD20 treatment and can be therapeutic in RCHOP resistant setting of DLBCL.

## Discussion

Here we show for the first time that combination of capivasertib and venetoclax has potential to provide broad activity in PTEN-wildtype and PTEN-deficient DLBCL preclinical models. This combination causes death of DLBCL cell lines in vitro and is highly efficacious in various lymphoma models in vivo. Concurrent dosing of capivasertib and venetoclax is necessary for optimal anti-tumoral activity and more importantly, we found that combination efficacy and disease control persists following adjustments to either dose level or frequency of venetoclax. Although reducing frequency of venetoclax treatment slightly reduced overall disease control in certain models, addition of rituximab restored maximal efficacy and promoted durable tumour regressions. Whilst directly targeting PI3K demonstrates clinical efficacy in haematological disorders, long-term treatment leads to toxicity issues thereby limiting therapeutic benefit [53]. AKT inhibitors offer an alternative approach to target downstream of PI3K, thus bypassing clinical tolerability issues, particularly in drug combination settings.

The PI3K-AKT pathway provides differential growth signals in DLBCL, however different subsets of disease are impacted by targeting different nodes in the pathway [7, 13]. Monotherapy treatment with PI3Kα/δ inhibitors reduces growth of ABC-DLBCL cell lines and tumour models, whilst monotherapy AKT inhibition in effective in PTEN null GCB DLBCL [7, 9]. Comprehensive inhibition of PI3K-AKT-mTOR signalling with the combination of mTORC1/2 and PI3Kβ inhibitors is effective in DLBCL cell lines of different subtypes [8, 29]. However, for the capivasertib venetoclax combination it was not possible to identify a specific subtype where the combination is more active, nonetheless broad anti-tumoral activity was seen in GCB-DLBCL models. PTEN protein loss is associated with GCB tumours [6, 7, 29] however, GCB-DLBCL PTEN wildtype models are also sensitive to combination treatment. Additionally, in the few ABC-DLBCL cells lines analysed, minimal activity was observed. It is however possible that combination activity is restricted to BCL2 positive lymphomas. Additional studies are necessary to refine patient selection strategies, although the data presented here suggest GCB-DLBCL would have the best outcomes with this combination. It is worth noting, activation of AKT signalling and expression of BCL2 fails to accurately predict combination sensitivity. For example, capivasertib activity is seen in the OCI-LY7 model that has lower levels of AKT signalling. Conversely, a lack of combination response could reflect other mutational drivers, or cellular factors that antagonise venetoclax activity. It is not clear what effect the combination of capivasertib and venetoclax has on other B-cell lineages, and whether it would mimic the B-cell depleting effects of rituximab.

AKT monotherapy activity in PTEN protein deficient solid and haematological tumour cell lines is expected, as well as in tumour with activating alteration in PIK3CA and AKT1. However, capivasertib can deliver monotherapy and combination activity beyond tumours with alterations in PTEN, PIK3CA or AKT1 [25, 53–55]. This is consistent with PI3K signalling regulating cell cycle and proliferation, AKT regulation of apoptosis, cholesterol biosynthesis, glucose metabolism, and gene expression [56–59]. This heterogeneity in dependency on different PI3K-AKT-mTOR signalling nodes in DLBCL suggests whilst the PI3K-AKT signalling pathway is critically important, the mechanism may vary between cell lines [7, 29].

Mechanistic studies in cell lines suggest that the combination drives cell death primarily through the intrinsic apoptotic pathway via MOMP formation, which engages caspase-8 and commonly initiates the extrinsic apoptosis pathway. This is accomplished without any intrinsic crosstalk between AKT signalling and intrinsic apoptosis pathways. A similar effect is observed in PTEN null breast cancer cell lines where AKT inhibition combines with the MCL-1 inhibitor AZD5991 to increase cell death independent of upstream PI3K pathway regulation [60]. Targeting PI3K, AKT or mTORC2 has been a proposed strategy to sensitize malignancies to BH3-mimetics [20, 22, 56, 61–64]. That capivasertib and venetoclax activity can be partially replicated with venetoclax monotherapy in galactose-substituted culture medium suggests that in part AKT inhibition may prime apoptosis through regulation of cellular metabolism. However, this does not wholly explain the combination response as venetoclax treatment in galactose-supplemented medium did not result in comparable changes in apoptotic caspase activity implying simply modulating glucose uptake is not sufficient to replicate the combination activity of capivasertib and venetoclax. Capivasertib monotherapy led to modest Mcl-1 degradation which was more evident in the combination with venetoclax. Again, this was not apparent following galactose supplementation. This may imply that AKT activity has some effect on or regulates Mcl-1 stability although the change in MCL-1 protein levels was modest and variable [22, 63]. However, we did not observe general regulation of BAD or BIM proteins or other regulators apoptosis. It is possible that modest reduction of Mcl-1 expression along with Bcl-2 inhibition may contribute to enhanced killing capacity by the combination and reduce a potential resistance mechanism [43, 65, 66].

Combining the PI3Kα/δ copanlisib and venetoclax has activity in haematological cancers, AML, MCL and DLBCL, however the mechanistic drivers of anti-tumoral activity remains elusive [9]. In AML cells combining the AKT inhibitor ipatasertib (GDC-0068) with venetoclax [67], resulted in cell death through BAX, with BAX deletion preventing cell death. This is possibly through direct phosphorylation of BAX by AKT [68] increasing association with the mitochondria, however in our study deleting BAX had minimal effect on combination sensitivity. Ipatasertib can induce apoptosis in cell lines through PUMA regulation however no evidence of an effect on PUMA in PTEN deficient tumour cells (data not shown) [69]. Interestingly, in PTEN deficient breast lines, synergy between the PI3Kβ inhibitor AZD8186 or capivasertib and AZD5991 (Mcl-1 inhibitor) depends on BAK and is differentiated from the effects in other studies and may indicate that sensitivity can be modulated through both BAK and BAX depending on context, perhaps through non-canonical mechanisms of regulation. An alternate hypothesis is that AKT pathway inhibition induces mitochondrial stress, priming loading of BAK (or BAX) complexes to the mitochondrial membrane with oligomerisation and lysis then triggered by the addition of the MCL1 inhibition. Finally modest cytochrome c leakage from the mitochondria either through PI3K pathway inhibition could be augmented using the combination of capivasertib and venetoclax.

In summary, we show here that combinations of capivasertib, venetoclax and rituximab are highly active in GCB DLBCL models. The combination activity can be achieved with flexible dosing of venetoclax allows potential to modified dose and schedule clinically to manage tolerability. Given the clinical challenges associated with PI3K inhibitors in haematological disease, capivasertib represents a novel approach to target the PI3K-AKT signalling axis with a different tolerability profile. Moreover, targeting AKT has the potential to combine with other relevant targeted therapies to improve response in subsets of DLBCL. The combination of capivasertib and venetoclax with addition of a CD20 antagonist is a potential therapeutic regimen for clinical evaluation in patients with relapsed/refractory DLCBL.

## Supplementary information

Supp Materials and Fig. Legends

Supp Figures

## Data Availability

Reagents and data can be accessed as appropriate by contacting simon.t.barry@astrazeneca.com.
