# Supplementary material for: Potent combination benefit of the AKT inhibitor capivasertib and the BCL-2 inhibitor venetoclax in diffuse large B cell lymphoma
Source: Leukemia. 2024 Sep 16;38(12):2663–74. doi: 10.1038/s41375-024-02401-9 (PMC11588655; doi:10.1038/s41375-024-02401-9)
Supplement: Supplementary file 1 — Supp Materials and Fig. Legends [file 41375_2024_2401_MOESM1_ESM.docx]

# **Willis et al Supplementary Tables and Figure legends**

# **Supplementary Tables**

## **Supplementary Table S1: Cell line culture conditions.**

| **Cell line** | **Base medium** | **Supplier** | **Cat. No.** | **Heat-inactivated FBS** | **Supplementation** |
| --- | --- | --- | --- | --- | --- |
| RIVA | RPMI-1640 | Gibco | 11835030 | 10% | 1% L-Glutamine |
| OCI-LY10 | IMDM | Gibco | 12440053 | 20% | 1% L-Glutamine, 50μM 2-βME |
| HBL-1 | RPMI-1640 | Gibco | 11835030 | 10% | 1% L-Glutamine |
| U2932 | RPMI-1640 | Gibco | 11835030 | 10% | 1% L-Glutamine |
| OCI-LY3 | RPMI-1640 | Gibco | 11835030 | 20% | 1% L-Glutamine |
| SUDHL2 | RPMI-1640 | Gibco | 11835030 | 10% | 1% L-Glutamine |
| OCI-LY1 | IMDM | Gibco | 12440053 | 20% | 1% L-Glutamine |
| ULA | IMDM | Gibco | 12440053 | 20% | 1% L-Glutamine, 50% Optimem |
| OCI-LY4 | IMDM | Gibco | 12440053 | 20% | 1% L-Glutamine, 50μM 2-βME |
| HT | RPMI-1640 | Gibco | 11835030 | 10% | 1% L-Glutamine |
| SUDHL6 | RPMI-1640 | Gibco | 11835030 | 15% | 1% L-Glutamine |
| OCI-LY8 | IMDM | Gibco | 12440053 | 20% | 1% L-Glutamine, 50μM 2-βME |
| OCI-LY7 | IMDM | Gibco | 12440053 | 20% | 1% L-Glutamine, |
| KARPAS-422 | RPMI-1640 | Gibco | 11835030 | 15% | 1% L-Glutamine |
| SUDHL5 | RPMI-1640 | Gibco | 11835030 | 15% | 1% L-Glutamine |
| SUDHL10 | RPMI-1640 | Gibco | 11835030 | 10% | 1% L-Glutamine |
| OCI-LY19 | RPMI-1640 | Gibco | 11835030 | 10% | 1% L-Glutamine |
| WSU-NHL | RPMI-1640 | Gibco | 11835030 | 10% | 1% L-Glutamine |
| BCV-1 | RPMI-1640 | Gibco | 11835030 | 20% | 1% L-Glutamine |
| DOHH2 | RPMI-1640 | Gibco | 11835030 | 10% | 1% L-Glutamine |
| Pfeiffer | RPMI-1640 | Gibco | 11835030 | 10% | 1% L-Glutamine |
| WILL2 | RPMI-1640 | Gibco | 11835030 | 20% | 1% L-Glutamine |
| WILL1 | RPMI-1640 | Gibco | 11835030 | 20% | 1% L-Glutamine |
| U2940 | RPMI-1640 | Gibco | 11835030 | 10% | 1% L-Glutamine |
| TMD8 | MEM-α | Gibco | 41061029 | 10% | 1% L-Glutamine |
| WSU-DLCL2 | RPMI-1640 | Gibco | 11835030 | 10% | 1% L-Glutamine |
| SUDHL-4 | RPMI-1640 | Gibco | 11835030 | 10% | 1% L-Glutamine |

## **Supplementary Table S2: List of Primary Antibodies.8**

| **Antibody** | **Supplier** | **Cat. No.** | **Dilution^1^** |
| --- | --- | --- | --- |
| Phospho-Akt (Ser473) | Cell Signaling Technology | CST9271 | 1:1000 |
| Phospho-Akt (Thr308) (C31E5E) | Cell Signaling Technology | CST2965 | 1:1000 |
| Phospho-PRAS40 (Thr246) (D4D2) XP | Cell Signaling Technology | CST13175 | 1:1000 |
| Cleaved PARP (Asp214) (D64E10) XP | Cell Signaling Technology | CST5625 | 1:1000 |
| Cleaved Caspase-3 (Asp175) (5A1E) | Cell Signaling Technology | CST9664 | 1:1000 |
| Cleaved Caspase-9 (Asp315) (D8I9E) | Cell Signaling Technology | CST20750 | 1:1000 |
| Caspase-8 (1C12) | Cell Signaling Technology | CST9746 | 1:1000 |
| Mcl-1 (D35A5) | Cell Signaling Technology | CST5453 | 1:1000 |
| Bak (D4E4) | Cell Signaling Technology | CST12105 | 1:1000 |
| Bax | Cell Signaling Technology | CST2772 | 1:1000 |
| Bcl-2 (D55G8) | Cell Signaling Technology | CST4223 | 1:1000 |
| Phospho-FoxO1 (Thr24)/FoxO3a (Thr32) | Cell Signaling Technology | CST9464 | 1:1000 |
| Phospho-GSK-3β (Ser9) | Cell Signaling Technology | CST9336 | 1:1000 |
| Phospho-S6 Ribosomal Protein (Ser235/236) | Cell Signaling Technology | CST2211 | 1:1000 |
| Phospho-4E-BP1 (Ser65) | Cell Signaling Technology | CST9451 | 1:1000 |
| Bcl-xL (54H6) | Cell Signaling Technology | CST2764 | 1:1000 |
| Phospho-p44/42 MAPK (Erk1/2) (Thr202/Tyr204) (D13.14.4E) XP | Cell Signaling Technology | CST4370 | 1:1000 |
| GAPDH (14C10) | Cell Signaling Technology | CST2118 | 1:2000 |
| MEK1/2 | Cell Signaling Technology | CST8727 | 1:1000 |
| AIF | Cell Signaling Technology | CST5318 | 1:1000 |
| Cytochrome C | Abcam | AB65311 | 1:1000 |
| Vinculin (VLN01) | Invitrogen | MA5-11690 | 1:2000 |

^1^ All antibodies were diluted in 5% milk in TBS-T for western blot analysis.

## **Supplementary Table S3. List of single-guide RNAs (sgRNA) for CRISPR/Cas9 gene editing.**

| **Name** | **Sequence** |
| --- | --- |
| Hs.Cas9.BAK1.1.AA | mG*mG*mA* rArCrU rCrUrG rArGrUrCrArU rArGrC rGrUrG rUrUrU rUrArGrArGrC rUrArG rArArA rUrArG rCrArArGrUrU rArArA rArUrA rArGrG rCrUrArGrUrC rCrGrU rUrArU rCrArA rCrUrUrGrArA rArArA rGrUrG rGrCrA rCrCrGrArGrU rCrGrG rUrGrC mU*mU*mU* rU |
| Hs.Cas9.BAK1.1.AB | mG*mU*mU* rUrGrA rGrArG rUrGrGrCrArU rCrArA rUrUrG rUrUrU rUrArGrArGrC rUrArG rArArA rUrArG rCrArArGrUrU rArArA rArUrA rArGrG rCrUrArGrUrC rCrGrU rUrArU rCrArA rCrUrUrGrArA rArArA rGrUrG rGrCrA rCrCrGrArGrU rCrGrG rUrGrC mU*mU*mU* rU |
| Hs.Cas9.BAX.1.AA | mA*mC*mU* rCrGrG rArArA rArArGrArCrC rUrCrU rCrGrG rUrUrU rUrArGrArGrC rUrArG rArArA rUrArG rCrArArGrUrU rArArA rArUrA rArGrG rCrUrArGrUrC rCrGrU rUrArU rCrArA rCrUrUrGrArA rArArA rGrUrG rGrCrA rCrCrGrArGrU rCrGrG rUrGrC mU*mU*mU* rU |
| Hs.Cas9.BAX.1.AB | mA*mC*mG* rGrCrA rArCrU rUrCrArArCrU rGrGrG rGrCrG rUrUrU rUrArGrArGrC rUrArG rArArA rUrArG rCrArArGrUrU rArArA rArUrA rArGrG rCrUrArGrUrC rCrGrU rUrArU rCrArA rCrUrUrGrArA rArArA rGrUrG rGrCrA rCrCrGrArGrU rCrGrG rUrGrC mU*mU*mU* rU |

**Supplementary Table 4. Dosing schedule for capivasertib and venetoclax.**

| **Treatment**  **Schedules** | **Day 1** | **Day 2** | **Day 3** | **Day 4** | **Day 5** | **Day 6** | **Day 7** |
| --- | --- | --- | --- | --- | --- | --- | --- |
| **Capivasertib**  **130 mg/kg**  **BID 10/14** | **X** | **X** | **X** | **X** |  |  |  |
| **Venetoclax**  **100 mg/kg**  **QD** | **X** | **X** | **X** | **X** | **X** | **X** | **X** |
| **Rituxan**  **10 mg/kg**  **2QW** | **X** |  |  | **X** |  |  |  |
| **Capivasertib**  **130 mg/kg**  **BID 10/14**  **+**  **Venetoclax**  **100 mg/kg**  **QD** | **X** | **X** | **X** | **X** |  |  |  |
|  | **X** | **X** | **X** | **X** | **X** | **X** | **X** |
| **Capivasertib**  **130 mg/kg**  **BID 10/14**  **+**  **Venetoclax**  **100 mg/kg**  **QD** | **X** | **X** | **X** | **X** |  |  |  |
|  | **X** | **X** | **X** | **X** |  |  |  |
| **Capivasertib**  **130 mg/kg**  **BID 10/14**  **+**  **Venetoclax**  **100 mg/kg**  **QD** | **X** | **X** | **X** | **X** |  |  |  |
|  |  |  |  |  | **X** | **X** | **X** |
| **Capivasertib**  **130 mg/kg**  **BID 10/14**  **+**  **Venetoclax**  **100 mg/kg 2QW** | **X** | **X** | **X** | **X** |  |  |  |
|  | **X** |  | **X** |  |  |  |  |
| **Capivasertib**  **130 mg/kg**  **BID 10/14**  **+**  **Venetoclax**  **100 mg/kg QW** | **X** | **X** | **X** | **X** |  |  |  |
|  | **X** |  |  |  |  |  |  |
| **Capivasertib**  **130 mg/kg**  **BID 10/14**  **+**  **Rituxan**  **10 mg/kg**  **2QW** | **X** | **X** | **X** | **X** |  |  |  |
|  | **X** |  |  | **X** |  |  |  |
| **Capivasertib**  **130 mg/kg**  **BID 10/14**  **+**  **Venetoclax**  **100 mg/kg QW**  **+**  **Rituxan**  **10 mg/kg**  **2QW** | **X** | **X** | **X** | **X** |  |  |  |
|  | **X** |  |  |  |  |  |  |
|  | **X** |  |  | **X** |  |  |  |
| **Capivasertib**  **130 mg/kg**  **BID 10/14**  **+**  **Venetoclax**  **100 mg/kg 2QW**  **+**  **Rituxan**  **10 mg/kg**  **2QW** | **X** | **X** | **X** | **X** |  |  |  |
|  | **X** |  | **X** |  |  |  |  |
|  | **X** |  |  | **X** |  |  |  |
| **Rituxan**  **10 mg/kg**  **2QW**  **+**  **Cyclophosphamide**  **25 mg/kg**  **SD**  **+**  **Doxorubicin hydrochloride**  **3 mg/kg**  **SD**  **+**  **Vincristine sulfate**  **0.25 mg/kg**  **SD**  **+**  **Prednisolone**  **0.5 mg/kg**  **QD x 5** | **X** |  |  | **X** |  |  |  |
|  | **X** |  |  |  |  |  |  |
|  | **X** |  |  |  |  |  |  |
|  | **X** |  |  |  |  |  |  |
|  | **X** | **X** | **X** | **X** | **X** |  |  |

**Supplementary Figure Legends**

**Supplementary Figure 1. Effect of capivasertib and venetoclax on caspase induction and proliferation in ABC-DLBCL cell lines.**

(A) 24h time course of caspase-3/7 activity in OCI-Ly10 (PTEN-wildtype; ABC) and U2932 (PTEN-null; ABC) cell lines (n=3/group) that were unresponsive to the either monotherapy treatment with capivasertib, venetoclax, staurosporine or the combination for capivasertib and venetoclax at the concentrations indicated. (B) Cell titer glo growth assay showing effect of capivasertib, venetoclax, the combination and staurosporine on proliferation of PTEN-null U2932 cells at drug concentrations at indicated time points.

**Supplementary Figure 2. Combination rapidly induces BAK/BAX-dependent caspase cleavage in responsive DLBCL cell lines.**

(A) Mitochondrial cytochrome c release following 2hr stimulation of SUDHL4 and WSU-DLCL2 with vehicle (DMSO), capivasertib, venetoclax and the combination at the indicated concentrations. (B) Time course of cells pretreated with Q-VD-OPH (50 µM) or vehicle and then dosed with compounds at 1, 2, and 4 hours. Cell models with CRISPR/Cas9-mediated genetic knockouts (KO) and double KO for BAK and BAX treated with compounds for 4 and 24 hours. ‘L’ indicates lane loaded with a protein ladder.

**Supplementary Figure 3. Body weight change following treatment with capivasertib, venetoclax and rituximab monotherapy and combination treatment groups.**

(A-C) Body weight change (%) of CB.17 SCID mice bearing the GCB-DLBCL cell line SUDHL4 xenograft tumours treated with capivasertib monotherapy, venetoclax monotherapy, and in combination in accordance with the doses and schedules indicated, also captured in Supplementary Table 1. (D-E) Body weight loss in NSG when both capivasertib and venetoclax were administered on a 3-day on/4-day off schedule at the doses indicated. (F-G) Body weight change (%) of CB.17 SCID mice bearing the GCB-DLBCL cell line SUDHL4 or SUDHL5 xenograft tumours treated with capivasertib monotherapy, venetoclax monotherapy, and in combination in accordance with schedules captured in supplementary Table 1. (H-J) Body weight change (%) of CB.17 SCID mice bearing the GCB-DLBCL cell line WSU-DLCL2 xenograft tumours following treatments as indicated (also shown in Supplementary Table 1) and RCHOP followed by a combination of capivasertib, venetoclax and rituximab at the doses indicated. Data are represented as mean body weights and standard error of the mean.
