# Supplementary figures and images for: Potent combination benefit of the AKT inhibitor capivasertib and the BCL-2 inhibitor venetoclax in diffuse large B cell lymphoma
Source: Leukemia. 2024 Sep 16;38(12):2663–74. doi: 10.1038/s41375-024-02401-9 (PMC11588655; doi:10.1038/s41375-024-02401-9)

Supplementary Figure 1.

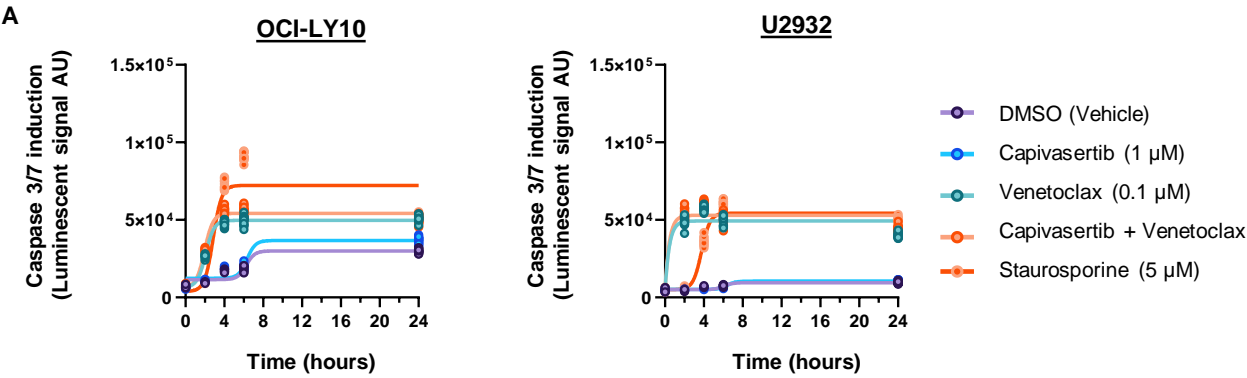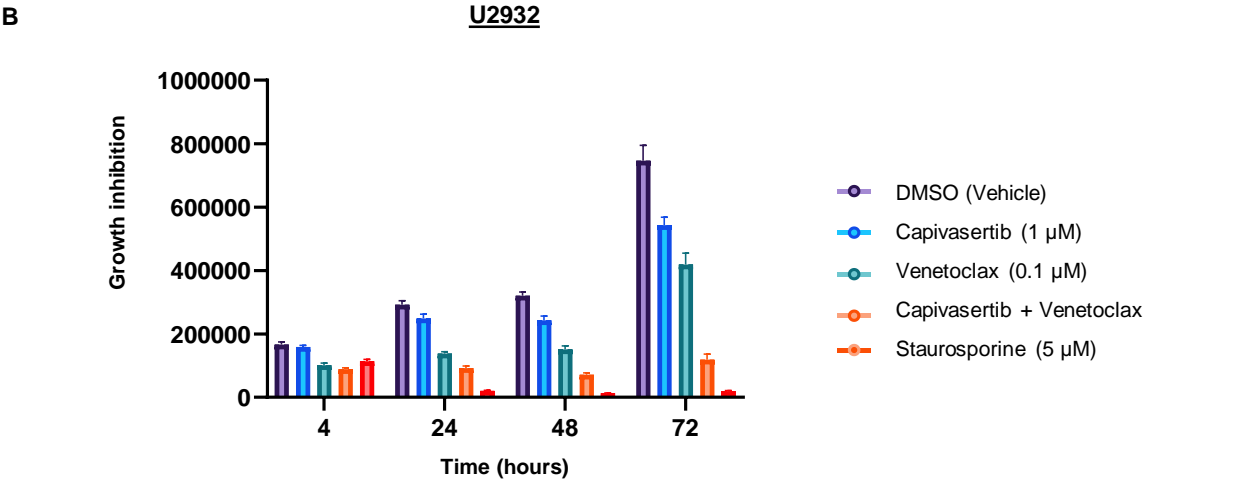

Supplementary Figure 2.

A

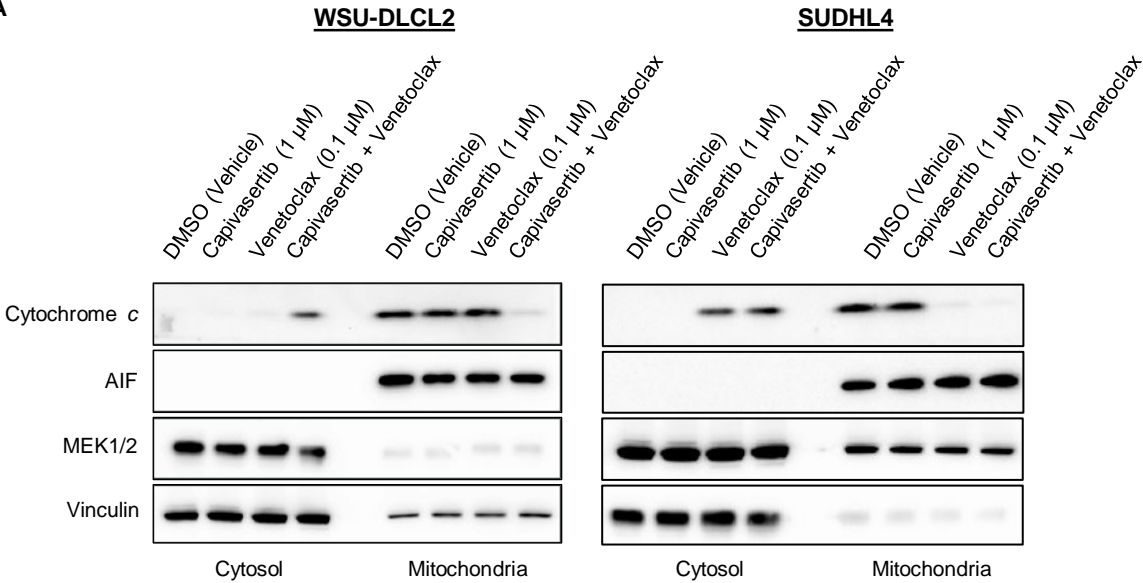

B

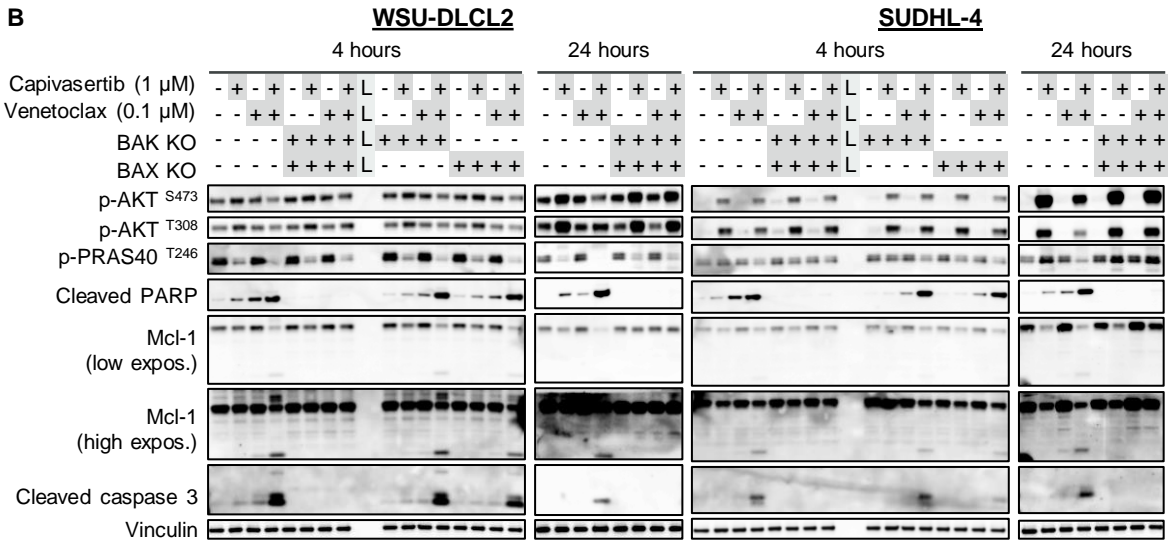

Supplementary Figure 3.

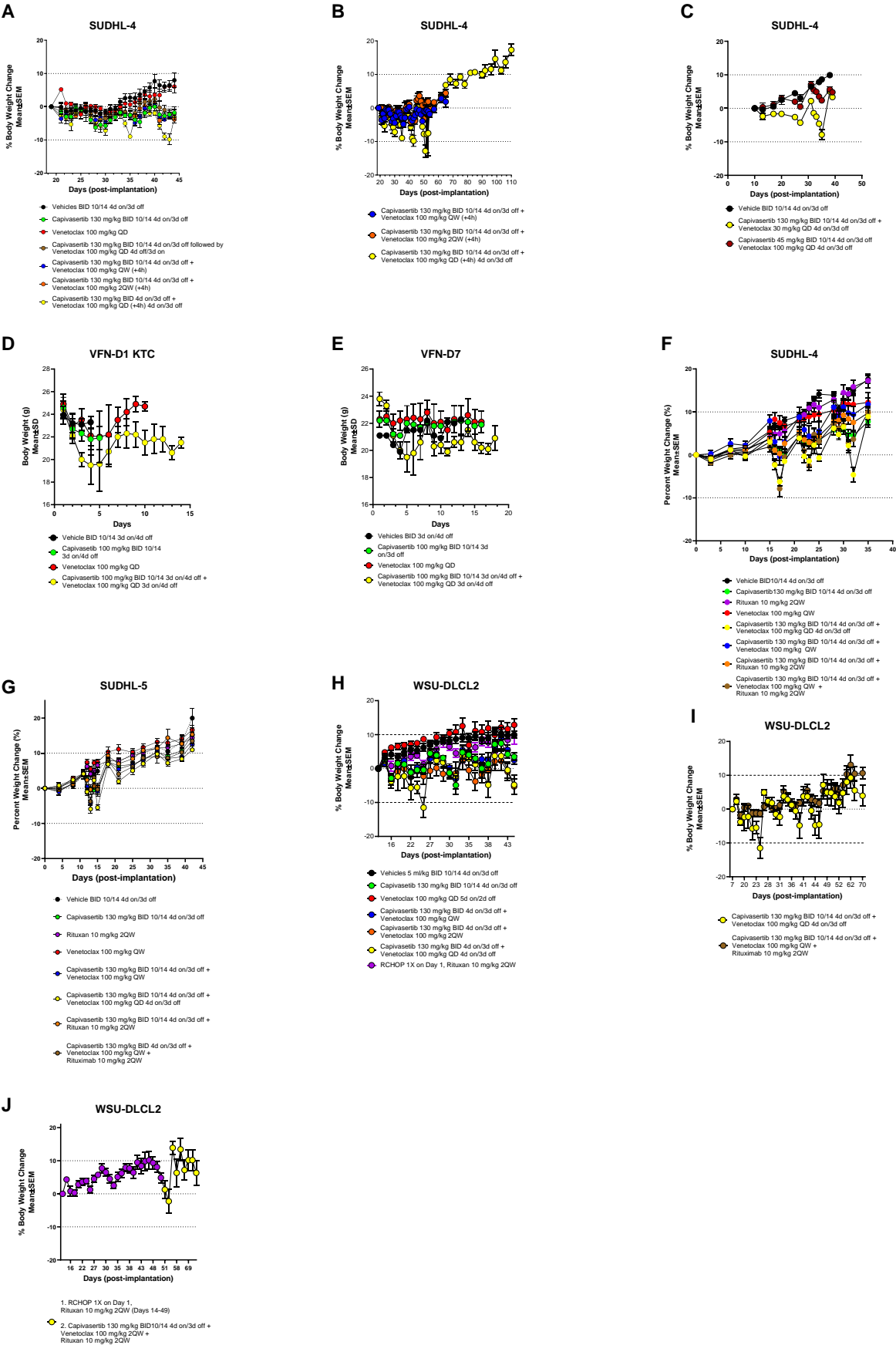

Supplement: Supplementary file 2 — Supp Figures [file 41375_2024_2401_MOESM2_ESM.pdf]
